# Compatible Solute Variation and Stress Adaptation in Native Qatari Plants: Focus on Proline

**DOI:** 10.3390/life16060951

**Published:** 2026-06-04

**Authors:** Roda F. Al-Thani, Bassam T. Yasseen

**Affiliations:** Department of Biological and Environmental Sciences, College of Arts & Sciences, Qatar University, Doha P.O. Box 2713, Qatar; ralthani@qu.edu.qa

**Keywords:** abiotic stress, biochemical roles, compatible solutes, physiological roles, salinity, halophytes, xerophytes, physiological roles

## Abstract

Osmolytes, including proline, soluble sugars, and glycine betaine (GB), are essential for plant adaptation to environmental stress. They contribute to osmotic adjustment, membrane stabilization, and the protection of cellular functions in arid and saline habitats. This study investigated major osmolytes (mainly proline and soluble sugars) in native Qatari plant species in natural field conditions and their physiological adaptation strategies. Significant interspecific variation indicated diverse mechanisms of stress acclimation. Although proline accumulation was common, it did not consistently correlate with salinity tolerance, which suggests that its accumulation may reflect stress-induced metabolic imbalance and adaptation rather than being a reliable indicator of resistance. Plant species, including crops and native plants such as *Avicennia marina*, *Halopeplis perfoliata*, *Limonium axillare*, *Tetraena qatarensis*, and *Ochradenus baccatus*, are presented as examples supporting this pattern. In contrast, the relative balance between soluble sugars and proline indicates coordinated carbon–nitrogen regulation that supports osmotic homeostasis and growth in fluctuating environmental conditions. Halophytic species exhibited distinct osmolyte profiles that highlight the potential role of additional compatible solutes (particularly GB) in stress adaptation. However, its occurrence and functional significance in these species have been insufficiently characterized. Given the predominance of C_3_ photosynthesis in Qatari flora, GB may also help mitigate photorespiratory stress in extreme conditions. The findings expand the current understanding of osmotic regulation in desert plants and highlight the potential of biotechnological approaches to enhance crop tolerance of harsh environments by manipulating compatible solutes.

## 1. Introduction

In response to environmental stresses such as drought, salinity, and extreme temperatures, plants accumulate compatible solutes that help to stabilize cellular structures and maintain metabolic homeostasis. Once the stress is relieved, these solutes are either remobilized or catabolized, after which nitrogen and other nutrients are released back into the cellular pool. The reclaimed resources are subsequently redirected toward the synthesis of essential macromolecules, including proteins and nucleic acids, which support cellular recovery and renewed growth.

As part of these adaptive responses, plants synthesize and accumulate various organic compounds in their tissues to alleviate osmotic stress [1,2]. These compounds are typically grouped into three major categories: (1) organic acids, (2) nitrogenous compounds, and (3) soluble sugars [3,4,5]. Representative examples include organic acids such as malate, citrate, and succinate; nitrogenous compounds such as proline, glutamate, ornithine, and glycine betaine (GB); and soluble sugars such as sucrose, glucose, fructose, and trehalose. These metabolites play essential roles in maintaining osmotic homeostasis by regulating cellular water balance and stabilizing proteins and membranes in the context of stress-induced damage, which strengthens plant resilience and adaptive capacity in adverse environmental conditions [6,7], such as extreme temperatures (both heat and freezing) [1,8,9].

Studies have revealed that the organic solute content of plants varies according to habitat conditions, and these solutes fulfil interchangeable physiological and biochemical roles during osmotic stress [10]. Osmoregulation in plant cells is achieved largely through the accumulation of compatible solutes such as proline, GB, soluble sugars, and essential inorganic ions such as potassium (K) [11]. These osmolytes also occur in associated microorganisms, including bacteria and fungi, where they perform similar protective and adaptive functions [1]. These mechanisms enable both plants and microbes to maintain cellular homeostasis and tolerate harsh environmental conditions [12]. This study examines the roles of compatible solutes in native Qatari plants—particularly xerophytes and halophytes—by exploring their types, functions, and patterns of accumulation across different plant tissues. The overall aim was to elucidate how these solutes contribute to stress tolerance in plants in diverse environmental conditions.

## 2. Adaptive Roles of Compatible Solutes in Native Plants

Among the major compatible solutes, proline, GB, and soluble sugars play central roles in plant responses to osmotic stress [13]. In both native plant species and their associated microorganisms, these solutes are involved in several essential functions:

(1) One significant role is osmotic adjustment, which maintains osmotic homeostasis through the accumulation of organic and inorganic solutes that balance osmotic potential between root tissues and the external environment [14]. Proline is especially important under drought and salinity stress because it lowers cellular osmotic potential, enabling continued water uptake and maintenance of turgor pressure [15,16]. By sustaining water absorption from dry or saline soils, proline supports long-term osmotic adjustment and prevents cellular dehydration [13]. GB and soluble sugars similarly contribute as compatible solutes that help maintain osmotic balance under stress. Effective osmotic regulation also depends on the differential distribution of solutes across cellular compartments. Plant cells vary in their ability to sequester solutes, making internal allocation crucial for osmotic homeostasis [15,17]. GB strongly correlates with leaf-cell sap osmotic potential in many species, highlighting its role in cytoplasmic balance [18], and is mainly localized in the cytoplasm, where it offsets vacuolar osmotic changes caused by inorganic ions such as Na^+^, Cl^−^, and Ca^2+^ [19]. This compartmentation prevents ionic disruption of metabolism and maintains cytoplasmic integrity and turgor [20]. Higher GB levels are also associated with reduced osmotic potential and improved water retention and stress tolerance [11]. Inorganic ions mainly accumulate in the vacuole, while compatible solutes such as proline, GB, selected sugars, and K^+^ accumulate in the cytoplasm to balance vacuolar ion loads without disrupting metabolism. These solutes do not interfere with enzyme activity even at high concentrations, thereby maintaining cellular hydration and biochemical stability. This coordinated regulation of internal solute distribution is known as osmoregulation and applies to plants, animals, and microorganisms [21,22]. Soluble sugars also contribute to osmotic adjustment, though their roles vary with species and conditions; sugars often increase under stress and can account for 30–50% of osmotic contribution in some glycophytes [3]. In contrast, halophytes rely more heavily on inorganic ions for osmotic balance, with organic solutes playing a smaller role [23,24].

(2) The protection and maintenance of intracellular structures, including organelles and enzyme systems, are essential for cell survival [1,15]. Compatible solutes such as proline help protect membranes and proteins from harmful high ion concentrations (e.g., Na, Cl, and heavy metals) and extreme temperatures by promoting proper protein hydration through preferential exclusion from protein surfaces, thereby stabilizing native folding. Proline also supports membrane integrity and cellular homeostasis under stress. Both proline and GB protect enzyme systems and organelles during heat stress [16]. These solutes act as multifunctional protectants, preserving the structural and functional integrity of enzymes, membranes, and organelles such as mitochondria, chloroplasts, the endoplasmic reticulum, and the cytoskeleton [25,26], making them highly effective natural protectants against temperature-induced damage.

(3) Act as key protective metabolites that serve as reserves of energy, reducing power, and metabolic intermediates following stress relief [25,27]. Their biosynthesis requires substantial metabolic investment in ATP (high-energy phosphate bonds) and NADPH, which provides reducing power. For instance, in the glutamate pathway, one molecule of proline requires one ATP and two NADPH, equivalent to about six ATP molecules, whereas the ornithine pathway uses one NADPH and approximately 2.5 ATP equivalents per molecule. Both pathways also draw on central metabolites needed for growth and essential physiological processes, including immune responses to biotic stress [28]. Although both operate in most plants, their relative contribution depends on species, stress intensity, and tissue nitrogen status; the glutamate pathway generally predominates under osmotic stress with limited nitrogen availability, while the ornithine pathway is favoured in nitrogen-rich conditions [29,30].

GB biosynthesis is also highly energy demanding, proceeding via choline → betaine aldehyde → GB, requiring significant metabolic input. Choline itself is costly to produce, being derived through serine → ethanolamine → phosphoethanolamine → choline, with multiple SAM-dependent methylation steps involving transfer of methyl groups (–CH_3_) from S-adenosyl-L-methionine. Some reactions require oxygen, along with reducing power in the form of NAD^+^/NADP^+^, and ATP consumption is substantial, with each SAM-dependent methylation costing ~3 ATP equivalents. Overall, GB synthesis requires approximately 12–15 ATP equivalents per molecule and one nitrogen atom per GB molecule [1,31,32].

Soluble sugars originate from photosynthetic carbon fixation and carbohydrate metabolism, with carbon skeletons generated through the Calvin cycle and associated pathways such as the pentose phosphate pathway (phosphogluconate pathway or HMP shunt). The roles of soluble sugars have been extensively described [3,33].

The accumulation of compatible solutes therefore imposes a significant metabolic cost under stress conditions, as large amounts of energy and carbon resources are redirected toward their synthesis and maintenance. While this investment supports cellular protection, osmotic adjustment, and stress tolerance, it can also limit resources available for growth, development, and yield, thereby reducing productivity under prolonged or severe stress. Nevertheless, the high energetic cost underscores their strong adaptive value, as proline, GB, and soluble sugars collectively contribute to osmotic balance, redox homeostasis, and the stabilization of proteins, membranes, and cellular structures under stress.

(4) Maintaining cellular turgor and hydration is a critical adaptive response to abiotic stress. Paleg and Aspinall [34] proposed that compatible solutes such as proline play a key role in regulating cellular water balance within cellular microstructures and across plasma membranes. This mechanism is essential for sustaining cell turgor, as proline interacts with hydrophobic residues on protein surfaces, thereby increasing the overall hydrophilicity of associated molecules. Such interactions enhance the stability of cellular components under salinity and water-deficit stress conditions [35,36].

(5) They can be utilized as a source of amino groups and energy, and their concentration declines rapidly once stress conditions are alleviated [34]. A major metabolic pathway involved is the rapid oxidation of proline to glutamate within the mitochondria as tissues regain turgidity. During this process, NADH is generated at the final stage of proline oxidation, as illustrated below:


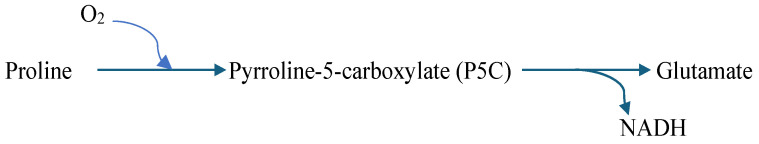


Energy 2 through the electron transport chain on the cristae of mitochondria. Glutamate is produced during proline oxidation and can be converted to α-ketoglutaric acid, which frees its amino group through a transamination process and allows it to enter the Krebs cycle. This is supported by the rapid evolution of CO_2_ when stressed plants regain access to water [33,37].

**Figure 1:**
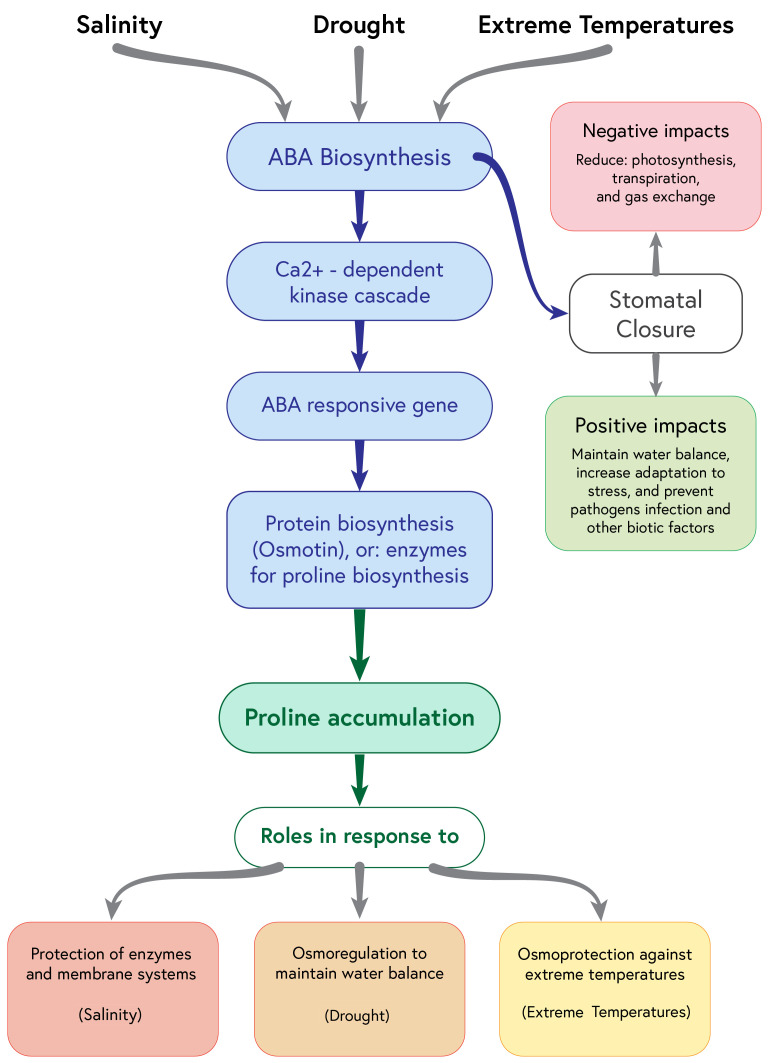
Physiological and biochemical responses of plants to abiotic stresses (salinity, drought, and extreme temperatures) mediated by ABA signalling.

(6) Can function as a sink for nitrogenous compounds that are released during net protein loss. Stress increases protein degradation while inhibiting protein synthesis. This leads to increased availability of nitrogen, which is subsequently directed toward proline formation [38,39].

(7) Proline as a compatible solute acts as an effective scavenger of reactive oxygen species (ROS), thereby helping to protect cells from oxidative stress. Proline can directly interact with several types of ROS, including hydroxyl radicals (•OH), singlet oxygen (^1^O_2_), and superoxide anions (O_2_•), which mitigates their damaging effects [40]. Through redox-mediated reactions, proline helps to neutralize reactive species and reduce their harmful effects, which protects key cellular macromolecules from oxidative damage, including lipids, proteins, nucleic acids, and membranes. Some compatible solutes influence protein solvation, act as an efficient scavenger of hydroxyl radicals, and contribute to membrane stabilization through their interactions with phospholipids [15,41,42].

(8) Other compatible solutes, such as GB, can sustain photosynthetic activity by maintaining chloroplast volume [43] or reducing photorespiration and increasing stomatal conductance [44], which stabilizes photosynthetic structures under stress [45]. Notably, GB supports photosynthesis in plants in the event of various types of environmental stresses by balancing the osmotic potential and preventing volume loss of cells and organelles. This is accomplished by maintaining optimal spacing and orientation of thylakoid membranes, which preserves efficient light reactions of photosynthesis. C_4_ plants are generally assumed to dominate saline and arid habitats due to their advantages in conditions of high temperature, intense light, drought, and salinity, but this trend does not apply to the Arabian Gulf. In the State of Qatar, C_3_ species constitute most of the native flora among all documented and recognized plant species.

(9) These solutes function as multifunctional signaling molecules that integrate metabolic, redox, and hormonal cues regulating gene expression, antioxidant defenses, and plant development [46]. Under severe environmental stress, proline accumulation is induced through abscisic acid (ABA)-mediated signaling pathways that activate proline biosynthesis genes [38,47]. ABA also promotes stomatal closure [48] and other physiological responses associated with stress adaptation (Figure 1). However, in some species, proline accumulation may indicate stress-induced damage rather than salinity tolerance [49].

In conclusion, the accumulation of compatible solutes in native plants and crops may indicate osmotic stress responses and contribute to adaptation under extreme environmental conditions.

### 2.1. Compatible Solutes as Indicators of Osmotic Stress Response

Controversial evidence from numerous plant species, including crops and native plants, suggests that the presence of compatible solutes may correlate with resistance, susceptibility, or visible injury under harsh environmental conditions. These observations are supported by histochemical, biochemical, and physiological studies conducted across a wide range of plant taxa [1,50]. Notably, variation in compatible solute profiles may represent a distinguishing feature among native species. For example, identifying the solutes accumulated by stress-resistant native plants, together with the genetic mechanisms regulating their synthesis and accumulation, may provide valuable insights for crop breeding programs. Such knowledge could contribute to the development of crops better adapted to environments increasingly affected by climate change and environmental degradation [50,51].

Early studies in halophytes showed that proline accumulation is closely linked to salt stress tolerance [52]. For instance, in *Triglochin maritima*, proline remains low under non-saline conditions but increases progressively with rising salinity. Similarly, coastal populations of *Armeria maritima* exhibit greater proline accumulation than inland populations, consistent with higher salt tolerance. Across many plant species, increased proline levels are consistently associated with stress exposure [53], making it a widely used physiological marker of environmental stress. Exogenous proline application has also been shown to enhance stress performance by improving biomass, photosynthesis, and gas exchange [54]. In salt-tolerant cultivars, higher endogenous proline under salinity is thought to help maintain cellular water balance and membrane stability [55]. Comparable patterns have been reported in eggplant and its wild relatives, where salt-resistant genotypes accumulate more proline under severe salinity [56]. Overall, these findings suggest that proline accumulation is a common response to salinity and is often associated with improved stress tolerance.

In contrast, other studies report that salt-sensitive plants may accumulate more proline than tolerant ones. For example, Yasseen [57] observed that certain salt-sensitive Mexican wheat varieties accumulated higher proline levels than tolerant types (Table 1). Several reviews similarly conclude that proline content does not consistently correlate with salinity or drought tolerance. In some cases, elevated proline has even been linked to stress susceptibility rather than adaptation, and it may act as an unreliable indicator in species such as soybean [58]. For instance, both susceptible (Bragg) and tolerant (Ransom) soybean cultivars show similar proline levels (0.4 μmol g^−1^ fresh weight) under mild salt stress (≤20 mM NaCl). However, at higher salinity (40–60 mM NaCl), Bragg accumulates substantially more proline (1.2–1.9 μmol g^−1^ fresh weight), whereas Ransom remains low, not exceeding 0.5 μmol g^−1^ even at 100 mM NaCl.

Proline accumulation was mainly observed in the susceptible cultivar under salt-induced physiological injury, suggesting it is not a reliable indicator of salt tolerance in soybean plants. Similarly, Sundaresan and Sudhakaran [59] reported in cassava that drought resistance did not correlate with proline accumulation. Under water stress, both the susceptible M-4 and resistant S-1315 showed increased leaf proline, but the rise was much greater in M-4 (≈25-fold) than in S-1315 (≈9-fold), alongside cultivar-specific differences in proline-metabolizing enzyme activity. Overall, these results support that proline accumulation is largely a consequence of stress rather than a determinant of stress tolerance or salt resistance [60].

### 2.2. Analysis of Stress Responses in Native Plants

Nature is characterized by remarkable biological diversity, reflected in the wide range of physiological activities and adaptive responses exhibited by living organisms. Although Qatar possesses a relatively small ecosystem, it is no exception to this diversity. Its native flora includes a wide variety of plant species adapted to harsh environmental conditions, and these adaptations are reflected in their metabolic and physiological responses. In particular, the role and variability of compatible solute accumulation in plant stress tolerance have been clearly demonstrated among native plants, including halophytes, xerophytes, and xero-halophytes. For example, many halophytes accumulate lower levels of proline in their natural habitats than xerophytes and xero-halophytes (Table 2). Moreover, halophytes accumulate a diverse range of compatible solutes that contribute to osmotic adjustment and protection against environmental stress.

Halophytes accumulate a range of compatible solutes and inorganic ions that do not interfere with cytoplasmic metabolism, including proline, glycine betaine (GB), soluble sugars, and potassium [61,62]. However, organic osmolyte synthesis is energetically costly and requires multiple metabolic precursors, which explains why many halophytes preferentially use abundant environmental ions such as Na^+^, Cl^−^, Ca^2+^, and Mg^2+^ for bulk osmotic adjustment under saline conditions. These plants possess efficient systems for ion uptake, transport, compartmentation, and detoxification, mainly through vacuolar sequestration, while organic solutes primarily support cytosolic protection and stress mitigation. Because inorganic ions can accumulate in large amounts, they effectively maintain osmotic balance and sustain a favorable water potential gradient between plant and environment [23,63,64]. Relying on inorganic ions instead of synthesizing organic osmolytes offers several advantages to these plants: (1) Inorganic ions are readily available for uptake and do not require highly specialized structures or mechanisms. (2) Their uptake and translocation involve relatively low metabolic costs. (3) Dependence on inorganic ions for osmoregulation preserves photosynthates that would otherwise supply carbon skeletons and energy for growth-related biosynthesis. (4) Osmotic adjustment in roots through inorganic ions obviates the need to transport photosynthates from the shoots to roots, thereby avoiding additional metabolic expenditure [65,66,67]. Nevertheless, halophytes also synthesize diverse organic solutes to satisfy physiological demands, which allows them to fulfil protective and regulatory roles in varying environmental conditions, as documented in numerous studies [13]. The accumulation of organic solutes can also constrain productivity, as carbon and energy are diverted from growth-related processes such as photosynthesis, cell division, and structural development toward osmotic regulation. This internal resource trade-off limits biomass accumulation and yield in both halophytes and salt-exposed crops, presenting a challenge for agricultural productivity under saline conditions [68]. Despite these limitations, halophytes remain valuable sources of bioactive compounds [69,70,71] and genetic traits that can be used to improve crop tolerance to salinity [72].

Soluble sugars in plants, such as glucose, fructose, sucrose, maltose, and trehalose, also play essential roles in stress conditions [3]. They serve as energy sources and reserves, contribute to osmotic adjustment, and help stabilize cellular structures during stress. Their involvement in osmotic stress tolerance has been widely documented in crop plants, particularly during two critical developmental stages: seed germination and vegetative growth. Increased levels of soluble sugar improve water-stressed plants’ ability to tolerate dehydration by contributing to osmotic adjustment [3]. As compatible solutes, these sugars help to maintain cellular turgor by facilitating water uptake from the soil, thereby alleviating the adverse effects of drought on growth and survival [73,74]. Yasseen et al. [3] reported significant variation in compatible solute accumulation during seed germination under stress. Under mild osmotic stress, rapid degradation of storage reserves (starch, proteins, and lipids) increases metabolite availability, which supports cell division and seedling establishment. In contrast, high salinity suppresses reserve mobilization, limits metabolite supply, and constrains growth. Under such conditions, available resources are increasingly redirected toward the synthesis of compatible solutes, particularly proline, which may reduce the pool of soluble sugars available for germination and growth. Many of these protective functions overlap with those of proline. Therefore, the balance between sugar and proline accumulation reflects a coordinated carbon–nitrogen regulatory mechanism that allows plants to adjust osmotic regulation and growth in fluctuating stress conditions [36]. It is noteworthy that seeds with rich carbohydrate reserves, such as wheat and barley, accumulate proline by using carbon skeletons derived from soluble sugars and amino groups, which are supplied by the protein-rich aleurone layer. In contrast, seeds of fenugreek (*Trigonella foenum-graecum* L.) mobilize protein reserves to synthesize both sucrose and proline to support osmotic adjustment between the germinating seeds and the surrounding solution [75,76]. These observations suggest an exchange or shift in functional roles between proline and soluble sugars or nitrogen-containing compounds in key physiological and biochemical processes under osmotic stress [35,77]. In many plant systems, intracellular osmoregulation can be achieved primarily through the accumulation of either proline or soluble sugars. In such systems, particularly in cases of limited nitrogen availability, proline biosynthesis is largely supported by carbon derived from carbohydrate degradation [29]. During vegetative growth, plants depend on photosynthesis and associated metabolic pathways to generate the metabolites required for cellular maintenance. These processes produce soluble sugars and compatible solutes, such as proline, which contribute to osmotic adjustment under salinity and drought stress [78]. When a local Yemeni cultivar of fenugreek is subjected to water deficit, monosaccharide and proline concentrations increase during vegetative growth, whereas sucrose levels remain relatively constant. This coordinated metabolic response helps sustain cellular water status and protect intracellular structures [3,79]. Notably, the maintenance of stable sucrose concentrations likely supports continued carbon transport and allocation while contributing to osmotic balance, thereby reinforcing physiological stability in drought conditions.

Overall, although proline and glycine betaine are widely associated with stress tolerance, their precise roles remain debated. Proline accumulation may reflect either adaptive protection or stress injury, depending on context, whereas glycine betaine is more consistently linked to improved stress tolerance through osmo-protection and maintenance of cellular homeostasis. Compatible solute accumulation is therefore a complex, context-dependent strategy influenced by species, stress intensity, and developmental stage, requiring careful interpretation when assessing plant stress responses [80,81,82].

## 3. Case Studies on Compatible Solutes in Native Plants in Qatar

The climate of the Arabian Gulf region in general, and Qatar in particular, is classified as arid to semi-arid. The region is among the warmest in the world, and its soils are typically saline, with *EC_e_* values (the electrical conductivity of the saturated soil extract) reaching up to 200 dSm^−1^ [83,84]. However, some *rawdahs* exhibit low to moderate salinity levels that average around 4 dSm^−1^ [35,85].

In a study on 23 native plant species, including both xerophytes and halophytes, Abdel-Bari et al. [35] reported clear differences in soil water content and *EC_e_* in the species’ natural habitats (Table 3). In general, Qatari soils are characterized as dry and saline. The soil moisture content ranges from 4% to 71% field capacity, while soil salinity (*EC_e_*) varies from 4 dS m^−1^ to more than 200 dS m^−1^. *Rawdahs* tend to have mildly saline soils with relatively high moisture content, whereas other parts of the country—particularly sabkhas and coastal areas—exhibit very high salinity levels. Thus, natural habitats in Qatar are predominantly arid and saline across most regions of the country.

The vegetation in Qatar is dominated by obligate halophytes and facultative xerophytes/halophytes that are well adapted to this region’s extreme environmental conditions. This means that most of these plants are special in that they can survive in very salty and very dry environments. Obligate halophytes are plants that must live in salty soils, are fully adapted to high-salinity conditions, and cannot survive in normal non-salty soils. Facultative xerophytes/halophytes are plants that can also grow in less harsh environments.

Variations in soil salinity and moisture across the country are mirrored in the diversity of native plant species. These variations influence their physiological and biochemical processes and lead to the build-up of different inorganic and organic solutes. Plants commonly accumulate soluble organic compounds in response to environmental stresses [1,3,34,63], yet Qatari native species have varied ability to store particular solutes, including proline, GB, and soluble sugars [10,35,87,88,89]. This variability corresponds to their ecological strategies, which are shaped by characteristic morphological, anatomical, physiological, and biochemical traits [90,91].

The broader objective of research in this area is to identify traits that can be leveraged in modern biotechnology to improve plant adaptation—particularly in crops—to environmental challenges such as salinity, drought, and pollution [13]. Approaches such as genetic engineering and marker-assisted selection can be used to harness these naturally evolved adaptive mechanisms to strengthen crop performance and support global food security [92]. Studies and reports from the past two decades indicate that these solutes—particularly proline, soluble nitrogen compounds such as GB, and soluble sugars—can fulfil complementary or even interchangeable roles in a range of physiological and biochemical processes while under osmotic stress [10]. Differences in the concentrations of these solutes indicate that native plants differ in their capacity to accumulate organic solutes and to exchange functional roles (Table 4).

The plants under investigation exhibit varying capacities to accumulate proline. For example, *Ochradenus baccatus* showed the highest proline accumulation among all species, followed by *Tetraena qatarensis*, *Limonium axillare*, *Suaeda vermiculata*, *Salsola rosmarinus*, *Suaeda aegyptiaca*, and *Capparis spinosa*. Lower proline levels were recorded in *Anabasis setifera*, *Cocculus pendulus*, and *Avicennia marina*, while *Pulicaria crispa* exhibited the lowest concentration among all plants examined. Variability in proline accumulation in native plants can be attributed to several factors [93].

Differences in species-specific metabolic responses to their native environmental conditions help explain the observed variation [28]. Each species has unique metabolic adaptations that are shaped by its environment, which is why species do not all behave or function the same way [94]. Plant species vary in their capacities for the synthesis, degradation, and transport of proline, which are processes that support functions such as osmoregulation, stress resistance, and energy acquisition [29]. These capacities are controlled by distinct sets of enzymes and transporter proteins, which result in substantial differences in proline accumulation, even among species experiencing similar stress conditions, as shown in Table 4. Such variation reflects the unique biochemical machinery that each species uses for proline production, turnover, and intracellular transport. Because proline synthesis is energetically costly, stress-adapted species must balance this expense with their photosynthetic capacity and respiration rate, while the availability of nitrogen and carbon precursors further modulates accumulation.

The expression levels of key enzymes in these pathways also differ widely across plant taxa [95]. Additionally, species have evolved different levels of activation in proline biosynthetic pathways in response to stress, with some lineages exhibiting markedly stronger induction under adverse conditions [96]. The types and strength of abiotic stress (such as water stress in xerophytes) decrease osmotic potential, which induces higher proline production. On the other hand, salinity causes both osmotic stress and ion toxicity, which lead to proline production [97]. Temperature extremes, light intensity, and soil nutrient levels also affect proline levels [98].

Energy limitations are another important factor since considerable metabolic expenditure is required for active ion transport, ion removal, and the build-up of organic osmolytes [99]. As a result, although these compatible solutes are vital for stress tolerance, their synthesis and related ion-regulating activities impose a significant energetic burden on the plant. This diversion of energy can restrict growth and reduce productivity in agricultural systems [1,100]. Additionally, the plants may possess inherent adaptations to drought and salinity stress that lessen their dependence on energetically costly proline accumulation [101].

The developmental stage of a plant also affects its proline accumulation. Seedlings, for instance, often exhibit higher proline levels during growth because they tend to be more sensitive to environmental stress, whereas mature tissues generally display lower or more stable concentrations. In natural ecosystems, however, accurately and consistently determining the developmental stage of each individual plant is challenging and complicates efforts to study patterns of proline accumulation [102]. Tissue-specific osmotic adjustment is common in halophytes, and leaves often accumulate more proline than roots because they are exposed to greater transpiration-driven stress. In many Qatari succulent halophytes, such as *Limonium axillare*, *Suaeda* species, *Tetraena qatarensis*, and possibly others (Table 4), proline and other compatible solutes accumulate and are stored in specialized water-storing tissues, which improves tolerance to salinity and drought [35,103].

Differences in morphological and osmotic adjustment strategies influence how plants manage osmotic stress. Not all species rely on proline for osmo-protection, and many halophytes synthesize other compatible solutes such as GB, soluble sugars, and polyols, which reduces their dependence on proline for osmoregulation. Halophytes may also accumulate inorganic ions to achieve osmotic adjustment in harsh saline conditions. In contrast, xerophytes often develop morphological and anatomical adaptations that lessen their need for biochemical osmo-protectants and reduce the demand for proline accumulation [104].

Proline levels in plants are strongly shaped by their ecological and evolutionary history, and species that evolved in harsh or variable environments often accumulate higher amounts as part of their stress tolerance strategy. Even within the same species, native or locally adapted populations may show stronger proline responses because they regularly encounter environmental stresses. Microhabitat differences can further influence proline accumulation, which means that even plants growing close to one another may display distinct proline levels due to subtle variations in their immediate surroundings [3,15,50,102].

According to compatible-solute analyses, species in the family Amaranthaceae generally accumulate substantially less proline than what is typically expected for plants that are exposed to pronounced drought or salinity stress. However, some members of this family are halophytes and exhibit xerophytic traits that enable them to accumulate appreciable amounts of proline. For example, *Salsola rosmarinus* and *Suaeda* spp. can accumulate proline levels of up to 400 μgg^−1^ fresh weight. Species from other families, such as *Limonium axillare* (Plumbaginaceae), *Ochradenus baccatus* (Resedaceae), and *Tetraena qatarensis* (Zygophyllaceae), also accumulate high concentrations of proline, although among these species, only *L*. *axillare* is considered an obligate halophyte [35,51,105]. These observations indicate that proline accumulation is species-specific, as some plants have regulatory mechanisms that are highly responsive to environmental stresses.

In contrast, several xerophytic species do not accumulate substantial amounts of proline in water-stress or saline conditions. For instance, *Heliotropium bacciferum* and *Pulicaria* spp. display minimal proline accumulation in arid or saline environments but may rely on other compatible solutes to maintain osmotic balance, such as soluble sugars or possibly GB [3,106]. These findings support earlier observations indicating that native plants differ in the types and amounts of organic solutes that they accumulate depending on habitat conditions. Furthermore, such solutes can fulfil interchangeable physiological and biochemical roles during osmotic stress [10].

Other native Qatari flora may accumulate compatible solutes as an adaptive strategy to tolerate salinity and arid environments. Yasseen and Al-Thani have documented several of these species [12], and Table 5 summarizes these plants and their potential roles in the remediation of soils contaminated by salinity, petroleum hydrocarbons, and heavy metals. The table also highlights the reported or putative presence of compatible solutes in these species (particularly GB).

## 4. Glycine Betaine Accumulation in Native Qatari Plants

Glycine betaine (GB) is a quaternary ammonium compound belonging to a broader group of compatible osmolytes, including proline betaine, β-alanine betaine, choline-O-sulphate, and 3-dimethylsulphoniopropionate (DMSP) [31]. These compounds are widely distributed in nature and occur at relatively high concentrations in marine invertebrates, fish, and certain plant species compared with microorganisms and terrestrial vertebrates [152,153]. GB accumulation is frequently associated with halophytic and, to some extent, xerophytic plant species adapted to such conditions [154]. In many plant families, including Amaranthaceae, Chenopodiaceae, Gramineae, and Poaceae, GB accumulates at relatively high concentrations (>5–100 µmol g^−1^ DW), typically higher in shoots than in roots [155]. This distribution pattern reflects species-specific metabolic regulation and habitat adaptation strategies.

The biosynthesis of GB is a metabolically demanding process involving a series of enzymatic reactions that convert ethanolamine to choline and subsequently to GB [148]. Key precursors include glycine, serine, and formate, which are interconnected through one-carbon metabolism. Ethanolamine is produced from glycine via serine, with formate contributing one-carbon units during these conversions, before undergoing sequential methylation reactions to generate choline [156]. These metabolites are also associated with photorespiratory metabolism, linking GB biosynthesis to primary carbon metabolism [33]. Despite its high energetic cost, this pathway plays an important role in maintaining cellular osmotic balance and enhancing plant tolerance to salinity and drought stress [157]. The biosynthesis of GB may also be linked to photosynthetic metabolism in plants adapted to arid environments. Most native plant species in Qatar utilize the C3 photosynthetic pathway, whereas fewer species employ C4 or CAM pathways [86,158]. Because photorespiration is an inherent feature of C3 photosynthesis, intermediates generated during this process can contribute to GB biosynthesis, creating a metabolic connection between photorespiration and osmolyte production [1,72,80]. In addition to its role in carbon metabolism, photorespiration contributes to redox regulation and protection of the photosynthetic apparatus under stress conditions [33,159,160]. This integration of metabolic pathways may support GB-mediated stress adaptation in the predominantly C3 flora of Qatar. GB accumulation varies among plant species and is often associated with their capacity to withstand environmental stress. Both C3 and C4 plants can accumulate GB, although the extent of accumulation differs considerably among taxa and remains poorly characterized in many native species of the Arabian Gulf region [2,80,161]. Compared with other compatible solutes, such as proline, some species preferentially accumulate GB, highlighting its importance as an adaptive trait in arid and saline environments. Further investigation of GB distribution and regulation in native plants may improve our understanding of stress adaptation mechanisms and support efforts to enhance crop resilience under challenging environmental conditions [148,,162].

The native plants listed in Table 4 show substantial variations in their proline levels. Therefore, determining GB levels in these plants is essential to provide a more complete understanding of the role of these compatible solutes in the environmental conditions of the Arabian Gulf, particularly since soluble sugars may play only a limited role [2,6,80,126]. Table 5 shows that many plant species, including both C3 and C4 types, can accumulate GB in their tissues. Several species of Qatari flora have been identified as GB accumulators, which contribute to stress tolerance and improve resilience in native plants: *Atriplex* spp., *Capparis spinosa*, *Cleome* spp., *Frankenia pulverulenta*, *Halopyrum mucronatum*, *Haloxylon salicornicum*, *Ochradenus baccatus*, *Pulicaria* spp., *Salicornia* spp., *Salsola* spp., *Sporobolus spicatus*, *Suaeda* spp., *Tamarix* spp., and *Tetraena qatarensis*.

Accumulation of GB in these species may occur both constitutively and in response to environmental stimuli, as various stress conditions can induce its synthesis, such as salinity, drought, high temperatures, and pollution from petroleum hydrocarbons and heavy metals [1,109]. Therefore, examining this compatible solute in native plants in Qatar and other Arabian Gulf countries is essential to better understand its distribution and potential roles in the regional flora. To the best of our knowledge, GB has only been investigated in a limited number of native plant species in Qatar, which highlights the need for further studies to elucidate its functional significance in these plants.

## 5. Compatible Solutes and Molecular Adaptation

Considerable attention has been devoted to understanding the molecular adaptations that enable plants to survive extreme environmental conditions, particularly in halophytes and xerophytes native to arid regions. Yasseen and Al-Thani [84] described adaptive mechanisms in wild plants of Qatar and the Arabian Gulf, classifying them into water relations, solute regulation, and structural adaptations [64,72]. Environmental stress adversely affects plant metabolism, growth, and development [163,164], driving extensive research into the molecular basis of stress tolerance. Key mechanisms involved in stress adaptation include ion homeostasis systems such as H^+^-ATPases, Na^+^/H^+^ antiporters, and Na^+^/K^+^ transporters; genes regulating osmolyte accumulation and water balance, including aquaporins; and stress-related proteins such as dehydrins and osmotin. In addition, metabolic adjustments, including the transition to CAM photosynthesis, contribute to tolerance under harsh environmental conditions [64,165].

Recent studies have also identified genes and proteins linked to morphological, physiological, and biochemical adaptation processes [166,167]. Although molecular studies on compatible solute accumulation in plants of the Arabian Gulf region remain limited, evidence from recent decades indicates that abiotic stress and pollution can stimulate osmolyte accumulation, thereby enhancing tolerance to extreme environments. Al-Thani and Yasseen [1,168] discussed mechanisms underlying compatible solute accumulation in plants and emphasized the role of halophilic bacteria associated with halophytes in improving salt resistance. Current strategies to enhance plant resilience increasingly integrate genetic and biological approaches, which have proven effective in improving tolerance to extreme environmental conditions [12]. Advances in molecular biology and biotechnology have enabled the transfer of stress-resistant genes from halophilic bacteria into plants, resulting in transgenic lines capable of synthesizing compatible solutes and exhibiting improved abiotic stress tolerance [1]. Moreover, horizontal gene transfer (HGT) has been proposed as a potential mechanism for the movement of bacterial salt-resistance genes into plants [169,170]. Microorganisms support halophytes under saline and other abiotic stresses through a range of mechanisms, including biofilm and exopolysaccharide production, chemotaxis, phytohormone regulation, nitrogen fixation, and phosphate solubilization. They can also produce phytohormone-degrading enzymes and help regulate osmolyte accumulation, collectively enhancing plant stress tolerance [1]. As the last work further suggested that native plants and their microbial partners—especially those producing compatible solutes—can be leveraged through biotechnological approaches to improve cultivation and restore saline and arid ecosystems [72].

The transfer of salt-tolerance genes from selected microorganisms into native plants may enhance compatible solute production and improve resilience to multiple stresses, including salinity, petroleum hydrocarbon contamination, and heavy metal pollution. In addition, such genetic modifications may facilitate the synthesis of valuable bioactive compounds [171]. Salt-tolerant bacteria associated with native plants possess diverse protective mechanisms that mitigate the effects of severe abiotic stresses on their hosts. Consequently, the application of advanced molecular and genetic engineering technologies to develop transgenic plants capable of producing compatible solutes represents a promising strategy for improving plant tolerance to environmental stressors and polluted conditions [1,12,168,172,173].

## 6. Concluding Remarks and Future Perspectives

Variations in compatible osmolytes and their accumulation under stress conditions occur at multiple levels, including solute type, concentration, and differences among locations, habitats, and species. Extensive research conducted over the past fifty years has demonstrated that plant osmolyte concentrations fluctuate markedly in response to environmental conditions and stress severity. Consequently, many studies have yielded inconsistent or even contradictory findings, leading to differing interpretations among researchers. Despite this variability, there is a broad consensus that compatible solutes—such as proline, glycine betaine (GB), and various soluble sugars—play a critical role in plant survival under stress. These compounds enhance stress tolerance by stabilizing cellular structures and maintaining osmotic balance. Their accumulation in plant tissues, particularly in species naturally adapted to saline or drought-prone environments, is widely recognized as a key physiological strategy supporting plant adaptation and resilience under harsh conditions.

Modern biotechnological approaches should be further explored to develop crops with greater capacity to accumulate these osmolytes, thereby improving resilience and sustaining growth under adverse conditions. Genetic and biological strategies are promising tools to achieve this objective, particularly in the face of escalating global challenges such as population growth, environmental pollution, climate change, desertification, and increasing soil salinity. Genetic manipulation of plants is widely regarded as a potential solution for mitigating the adverse impacts of environmental stressors. Biological approaches have emerged as an effective means of addressing major environmental issues, including salinity stress and the contamination of ecosystems by petroleum hydrocarbons and heavy metals [1,8,172,173]. Such approaches should concentrate on native plants that can accumulate GB either through induction or constitutively, as this compatible solute provides effective protection against environmental stresses, including pollution resulting from human activities or wars.

## Figures and Tables

**Table 1:** Proline content in leaves of Mexican wheat cultivars differs in their salt resistance [57].

NaCl Treatment (dSm^−1^)	μmol g^−1^ FW	
	Cajeme (Salt Resistant)	Yecora (Salt Sensitive)
0	0.21	0.21
75	0.33	0.57
150	1.96	3.75

The reported means of proline concentration were calculated from four experimental observations.

**Table 2:** Proline content varies among five native Qatari plants with different ecological types [35].

Plant Species	Ecological Type	μgg^−1^ FW *
*Avicennia marina*	Halophyte: Mangroves	11–37
*Halopeplis perfoliata*	Halophyte: Sabkhas	56–281
*Limonium axillare*	Halophyte: Sabkhas and coastlines	103–810
*Tetraena qatarensis*	Xero-halophyte	419–1136
*Ochradenus baccatus*	Xerophyte	1277–1347

* The ranges are from six observations.

**Table 3:** Studied plant species, their corresponding ecological types, and selected physical characteristics in their natural habitats [35].

Plant Species	Family	Ecological Type	Soil Water Content (% FC) ^I,^*	*EC_e_* of the Soil (dSm^−1^) *
*Aeluropus lagopoides* (Monocot)	Poaceae	Xerophyte: A grass can live in saline habitats as well	40–66	45–50
*Anabasis setifera*	Chenopodiaceae	Facultative halophyte ^II^	12–43	71–198
*Arhrocenmum macrostachyum*	Amaranthaceae	Halophyte: It grows in coastal and inland salt marshes, alkali flats, and other habitats with saline soils	24–48	27–198
*Avicennia marina*	Acanthaceae	Halophyte: Mangrove, well adapted to coastal intertidal zones	Saturated soil	Seawater
*Capparis spinosa*	Capparaceae	Xerophyte: Its morphological and physiological features reflect its ecological type	9–12	4–5
*Caroxylon imbricatum* (previously *Salsola imbricata*)	Amaranthaceae	Xerohalophyte: A plant adapted to both drought and salinity; found in disturbed areas, saline soils, and coastal areas	11–16	150–195
*Cocculus pedulus*	Menispermaceae	Xerophyte	9–12	4–5
*Halocnemum strobilaceum*	Amaranthaceae	Halophyte: Lives in tidal zones and salt flats; grows in coastal and inland salt marshes, alkali flats, and other habitats with saline soils	34–61	12–198
*Halopeplis perfoliata*	Amaranthaceae	Halophyte: Found in highly saline sabkhas with sandy shelly soil	34–50	12–178
*Haloxylon salicornicum*	Amaranthaceae	Xerophyte: Found in stony soil overlain with wind-blown sand	9–12	80–90
*Heliotropium bacciferum*	Boraginaceae	Xerophyte with minor salt tolerance, not a halophyte	9–10	5–72
*Limonium axillare*	Plumbaginaceae	Halophyte: Found on coastline with saline shelly soil	32–43	12–198
*Ochradenus baccatus*	Resedaceae	Xerophyte: Found in disturbed areas	4–12	7–8
*Pulicaria crispa*	Asteraceae	Xerophyte: Found in low depressions and water catchment areas in sandy, slightly saline soil	5–8	4–6
*Pulicaria gnaphalodes*	Asteraceae	Xerophyte: Found in shallow depressions and rain pools with sandy stony soil; extremely woolly with pleasant smell	5–8	4–6
*Salsola soda*	Amaranthaceae	Halophyte: It thrives in saline environments and is found in salt marshes, coastal areas, and saline soils	43–71	>200
*Salicornia* spp. ^III^	Amaranthaceae	Halophytes: Found on flooded landward side of mangrove associated with *Salsola soda* and intertidal zones and sabkha depressions	43–71	>200
Salsola *rosmarinus* (Syn. *Seidlitzia rosmarinus*)	Amaranthaceae	Xerohalophyte; Can be regarded as both a halophyte and a xerophyte because it thrives in harsh, arid–saline desert habitats	14–30	52–81
*Sporobolus spicatus* (Monocot)	Poaceae	Halophyte: Commonly known as salt grass due to its ability to thrive in high-salinity environments, has salt gland structures to excrete extra salts, grows in arid regions and can tolerate drought conditions	32–44	107–128
*Suaeda aegyptiaca*	Amaranthaceae	Xerohalophyte, is highly adapted to environments with both high salinity and low water availability, found in depressions with high water tables and coastal areas	11–16	150–195
*Suaeda vermiculata*	Amaranthaceae	Halophyte and xerophyte (drought-tolerant plant): Found in moist saline soils and sabkhas	8–43	32–142
*Tetraena qatarensis* ^IV^	Zygophyllaceae	Xerohalophyte: Adapted to environments that are both dry and salty; many features should be discussed	10–46	12–187
*Ziziphus nummularia*	Rhamnaceae	Xerophyte: Is highly adapted to and primarily found in hot, arid, and dry regions (xeric habitats); found in *rawdahs* with depth and fine soil	9–12	4–5

^I^ FC: field capacity; ^II^ facultative halophyte: a plant that can grow in both saline and non-saline environments, although it is well adapted to high-salt conditions and does not require salt for its growth and survival; ^III^ *Salicornia* spp.: includes about three species, though their classification has not been clarified with well-known species. Abdel-Bari [86] has reported three species. *Salicornia macrostachya*, *S*. *perfoliate*, *S*. *strobilacea,* and *S*. *europaea* were included among the halophytes studied by Abdel-Bari et al. [35]. ^IV^ This plant is characterized as a xero-halophyte based on many factors. * The ranges are from six observations. N.B. Soil properties and the physiological and biochemical parameters listed in Table 3 and Table 4 were determined at the time of plant sampling.

**Table 4:** Plant water content, proline, total soluble sugars (TSS), and total soluble nitrogen (TSN) measured in shoot systems of selected native plant species in Qatar in natural habitat conditions [35].

Plant Species	Plant Water Content (%) *	Range of Proline (μgg^−1^ Fresh Weight) *	Range of TSS (mgg^−1^) DW *	Range of TSN (μgg^−1^) DW *
*Aeluropus lagopoides*	70–72	241–253	1.5–1.7	84–126
*Anabasis setifera* ^I^ (Syn. *Salsola setifera*)	72–76	21–54	1.6–2.0	81–92
*Arhrocenmum macrostachyum*	81–89	10–112	4.0–14.1	79–165
*Avicennia marina*	66–69	11–37	2.2–6.0	40–63
*Capparis spinosa*	62–68	131–302	No data	No data
*Caroxylon imbricatum* previously: *Salsola imbricata*	80–81	38–82	1.6–3.1	77–128
*Cocculus pedulus* ^II^	60–69	34–51	No data	No data
*Halocnemum strobilaceum*	71–80	19–73	1.8–2.5	48–146
*Halopeplis perfoliata*	78–88	56–281	2.9–3.5	46–88
*Haloxylon salicornicum*	70–78	91–119	No data	No data
*Heliotropium bacciferum*	69–76	151–192	10.6–16.6	73–140
*Limonium axillare*	64–72	103–810	4.7–8.0	67–169
*Ochradenus baccatus*	68–71	1277–1347	2.9–3.5	92–109
*Pulicaria crispa*	59–66	25–32	8.8–9.9	127–144
*Pulicaria gnaphalodes*	59–66	32–119	6.3–7.2	63–92
*Salicornia* spp. ^III^	80–87	55–172	No data	No data
*Salsola rosmarinus* (Syn. *Seidlitzia rosmarinus*)	84–88	161–327	2.8–4.7	77–119
*Salsola soda*	83–89	132–185	No data	No data
*Sporopolus spicatus* (Monocot)	58–66	40–147	3.3–3.4	195–236
*Suaeda aegyptiaca*	86–88	80–317	3.9–4.3	100–119
*Suaeda vermiculata*	77–82	96–409	6.7–19.8	104–161
*Tetraena qatarensis*	81–87	419–1136	2.9–4.3	85–190
*Ziziphus nummularia*	62–67	87–97	No data	No data

^I^ Facultative halophyte: a plant that can grow in both saline and non-saline environments, although it is well adapted to high-salt conditions and does not require salt for its growth and survival; this species was re-named as *Salsola setifera*. ^II^ More clarifications are needed for proline accumulation and other solutes. ^III^ See Table 3 for more clarification. TSS: Total soluble sugars; TSN: total soluble nitrogen. * The ranges are from six observations.

**Table 5:** List of some native Qatari plants, including halophytes and xerophytes, their remediation role, and presence of compatible solutes.

Species/Family	Phytoremediation Activity	Compatible Solute Accumulation ^#^	Remarks and Observations	References
*Aeluropus lagopoides* * Fam.: Poaceae	Petroleum hydrocarbons, heavy metals such as Cd and Pb	Possible accumulation	C4 grass, exhibits some xerophytic adaptations	[12,107,108]
*Arhrocenmum* spp. *, 4 species, Amaranthaceae	Remediate heavy metals; petroleum hydrocarbons require further investigation	Little proline was detected; higher levels of soluble sugars and nitrogen were observed, and GB accumulation is possible	C3 plant, some adaptations to desert habitats	[12,18]
*Atriplex* spp. *** 2 species *Amaranthaceae*	Heavy metals: Cd, Cu, Ni, Pb, and Zn; petroleum hydrocarbons need further investigation	Accumulate proline, GB, and soluble sugars in their natural habitats; ABA increases under salt stress	C3 and C4 plants, xerophytes and halophytes are recognized among this genus	[109,110]
*Avicennia marina* * Acanthaceae	Heavy metals Cd, Co, Cr, Cu, Fe, Ni, and Zn and petroleum hydrocarbons	Little proline was detected; higher levels of soluble sugars were observed; GB needs further confirmation	C3 plant, mangrove tree	[12,111]
*Capparis spinosa* ** Capparaceae	Accumulates heavy metals and degrades petroleum hydrocarbon	Accumulates proline, and QAC; needs further investigation for GB and soluble sugars	C3 plant, might resist salinity to a certain level	[35,112]
*Cleome* spp. ** Cleomaceae (formerly Capparaceae)	Remediates heavy metals such as Cd and Cu, needs confirmation	Accumulates proline, GB, and soluble sugars in natural habitats	C3 or C4 plants, depending on species, accumulates some fatty acids for detoxification	[12,,113]
*Cocculus pendulus* ** Menispermaceae	No information, needs to be tested	Little proline was detected, might accumulate GB, needs further investigation for soluble sugars	C3 plant	[35,45,114]
*Cressa cretica* * Convolvulaceae	Remediates some heavy metals, possibly remediates petroleum hydrocarbons	Accumulates proline, needs further investigation for GB and soluble sugars	C4 plant	[12,115]
*Cyperus* spp. *, **, ^$^, 3 species Cyperaceae	Remediates many heavy metals such as Al, Cd, Co, Cr, Cu, Fe, Hg, Mn, Ni, Pb, and Zn (phyto-stabilization of Ni), petroleum hydrocarbons	Accumulate proline, possibly GB, and soluble sugars	Mainly C4, some C3, species in Qatar are salt tolerant, xerophytes, and mesophytes	[12,116]
*Frankenia pulverulenta* * Frankeniaceae	Remediates heavy metals such as Cd, Cr, Cu, Ni, Sr, and Zn, petroleum hydrocarbons	Accumulates compatible solutes: proline, GB, and soluble sugars	C3 plant, found in moist saline soil	[12,117,118]
*Halocnemum strobilaceum* * Amaranthaceae	Accumulates heavy metals, such as Cd, Cu, Fe, Mn, Ni, Pb, and Zn; needs further studies for petroleum hydrocarbons	Accumulates compatible solutes such as proline and soluble sugars	C4 plant, obligate halophyte, might accumulate GB	[1,12,119]
*Halodule uninervis* * Hydrocharitaceae	Accumulates heavy metals such as Cu, Fe, Ni, and Pb; phytoremediates petroleum hydrocarbons	Possible accumulation of compatible solutes; needs to be tested	C3 plant, marine hydrophyte seagrass, halophyte	[12,77,120]
*Halopeplis perfoliata* * Amaranthaceae	Remediates some heavy metals and may help remediate petroleum hydrocarbons	Accumulates proline and possibly other compatible solutes such as GB and soluble sugars; needs confirmation	C4 plant, succulent	[1,35,121]
Halopyrum mucronatum * Poaceae	Some heavy metals are accumulated; considered as bioindicator for Cr, Fe, Pb, and Zn; needs to be tested for phytoremediation of petroleum hydrocarbons	Accumulates GB and possibly other compatible solutes	C4 plant, coastal halophytic grass	[1,3,108,109]
*Haloxylon salicornicum* ** Amaranthaceae	Accumulates heavy metals such as Cu, Fe, Mn, and Zn; possibly phytoremediates petroleum hydrocarbons	Little proline was detected; accumulates GB	C4 plant, native to arid and semi-arid desert region	[12,88,109,122]
*Heliotropium* spp., **, 8 species, Boraginaceae	Possible role in phytoremdiation of organic pollutants and heavy metals	Little proline was detected; large amounts of soluble sugars accumulated; testing needed for GB	C3 plants, can be considered as semi-xerophyte, might be found in coastal saline soil	[3,35,123,124,125]
*Juncus rigidus* ***, Juncaceae	Phytoremediate organic compounds, heavy metals, and saline soil	Might accumulate compatible solutes; further investigation is needed	C3 plant, shows some xerophytic features	[126,127]
*Limonium axillare* * Plumbaginaceae	Remediates saline soil and heavy metals	Proline and soluble sugars accumulate, some relatively compatible solutes such as β-alanine betaine and choline-O-sulphate	C3 plant, found in coastal saline habitats, moist lands	[12,105,128]
*Mesembryanthemum* spp. ***, 2 species, Aizoaceae	Could be used for phytoremediation of contaminated soil; needs testing	Might accumulate compatible solutes under stress; needs testing	CAM plant	[129,130]
*Ochradenus baccatus* **, Resedaceae	Needs testing for petroleum hydrocarbon phytoremediation	Accumulates compatible solutes such as proline, soluble sugars, and possibly GB	C3 plant	[1,3,35]
*Polypogon monspeliensis* *, Poaceae	Accumulates heavy metals; phytoremediates petroleum hydrocarbons and saline soil	Needs testing for compatible solutes	C3 plant, possible accumulator of GB	[1,12,131,132,133]
*Pulicaria* spp. ** 4 species, Asteraceae	Possible candidates for petroleum hydrocarbons; need testing	Little proline was detected; accumulate significant amounts of soluble sugars; possible accumulation of GB; needs testing	C3 plants, tolerate salinity	[35,45,134]
*Salicornia* spp. *, 3 species, Amaranthaceae	Possible phytoremediation candidates for petroleum hydrocarbon and heavy metals; need further investigation	Very strong evidence that these species accumulate compatible solutes such as proline, soluble sugars, and GB; further investigation is needed	C3 plants, obligate halophytes, biochemical adaptation; these species have several applications in the food, feed, pharmaceutical, cosmetics, and bioenergy sectors	[35,135,136,137,138]
*Salsola* spp., *, **, 9 species, Amaranthaceae	Possible role in phytoremediation, especially heavy metals and saline soils; some species remediate heavy metals such as B, Cu, Mn, and Se; further investigations are needed	Accumulate proline and soluble sugars and might accumulate other compatible solutes such as GB; needs testing	C4 plants, including halophytes and xerophytes; some bioactive uses were reported; industrial and nutritional values	[35,139,140,141,142,143]
*Sporobolus spicatus* * (Monocot), Poaceae	Phytoremediates petroleum hydrocarbons, heavy metals, and saline soils	Accumulates compatible solutes such as proline and soluble sugars; GB might have a role in salt tolerance	C4 plant; proven efficient in controlling polluted soils	[12,144,145]
*Suaeda* spp. * 7 species, Amaranthaceae	Phytoremediates heavy metals and possibly petroleum hydrocarbons; testing is needed	Accumulates proline, soluble sugars, and possibly GB; some species were proven to accumulate GB	C3 and C4 plants are found in the genus, application of GB might improve plant growth in saline conditions	[35,92]
*Tamarix* spp. ***, 5 species, Tamaricaceae	Phytoremediates heavy metals and polycyclic aromatic hydrocarbons	Accumulate significant amounts of compatible solutes such as proline and soluble sugars; GB accumulation needs confirmation	C3 plants	[12,146,147]
*Tetraena qatarensis* ***, Zygophyllaceae	Accumulates heavy metals; possibly phytoremediates petroleum hydrocarbons	Accumulates significant amounts of proline and less soluble sugars; GB accumulation needs confirmation	C4 plant	[35,45,148,149]
*Teucrium polium* **, Lamiaceae	Accumulates heavy metals such as Co and Ni; phytoremediates petroleum hydrocarbons	Accumulates compatible solutes such as proline and soluble sugars	C3 plant	[12,150,151]

* Halophyte, ** xerophyte, and *** xerohalophyte. ^#^ See Table 4 for proline and soluble sugars. ^$^ Mesophyte.

## Data Availability

The original contributions presented in this study are included in the article, and further inquiries can be directed to the corresponding author.
